# Understanding the impact of exposure to adverse socioeconomic conditions on chronic stress from a complexity science perspective

**DOI:** 10.1186/s12916-021-02106-1

**Published:** 2021-10-12

**Authors:** Loes Crielaard, Mary Nicolaou, Alexia Sawyer, Rick Quax, Karien Stronks

**Affiliations:** 1 Department of Public and Occupational Health, Amsterdam UMC, University of Amsterdam, Amsterdam Public Health Research Institute, Meibergdreef 9, Amsterdam, 1105 AZ The Netherlands; 2 Institute for Advanced Study, University of Amsterdam, Amsterdam, 1012 GC The Netherlands; 3 Centre for Urban Mental Health, University of Amsterdam, Amsterdam, 1012 GC The Netherlands; 4 Computational Science Lab, University of Amsterdam, Amsterdam, 1098 XH The Netherlands

**Keywords:** Socioeconomic status, Causal loop diagram, Chronic stress, Feedback loops

## Abstract

**Background:**

Chronic stress increases chronic disease risk and may underlie the association between exposure to adverse socioeconomic conditions and adverse health outcomes. The relationship between exposure to such conditions and chronic stress is complex due to feedback loops between stressor exposure and psychological processes, encompassing different temporal (acute stress response to repeated exposure over the life course) and spatial (biological/psychological/social) scales. We examined the mechanisms underlying the relationship between exposure to adverse socioeconomic conditions and chronic stress from a complexity science perspective, focusing on amplifying feedback loops across different scales.

**Methods:**

We developed a causal loop diagram (CLD) to interpret available evidence from this perspective. The CLD was drafted by an interdisciplinary group of researchers. Evidence from literature was used to confirm/contest the variables and causal links included in the conceptual framework and refine their conceptualisation. Our findings were evaluated by eight independent researchers.

**Results:**

Adverse socioeconomic conditions imply an accumulation of stressors and increase the likelihood of exposure to uncontrollable childhood and life course stressors. Repetition of such stressors may activate mechanisms that can affect coping resources and coping strategies and stimulate appraisal of subsequent stressors as uncontrollable. We identified five feedback loops describing these mechanisms: (1) progressive deterioration of access to coping resources because of repeated insolvability of stressors; (2) perception of stressors as uncontrollable due to learned helplessness; (3) tax on cognitive bandwidth caused by stress; (4) stimulation of problem avoidance to provide relief from the stress response and free up cognitive bandwidth; and (5) susceptibility to appraising stimuli as stressors against a background of stress.

**Conclusions:**

Taking a complexity science perspective reveals that exposure to adverse socioeconomic conditions implies recurrent stressor exposure which impacts chronic stress via amplifying feedback loops that together could be conceptualised as one vicious cycle. This means that in order for individual-level psychological interventions to be effective, the context of exposure to adverse socioeconomic conditions also needs to be addressed.

**Supplementary Information:**

The online version contains supplementary material available at 10.1186/s12916-021-02106-1.

## Background

Chronic stress is defined as the repeated occurrence of the stress response, which involves cognitive, affective and biological reactions induced by a stressor [1], over an extended period of time, i.e. several hours per day during weeks to months [2]. Chronic stress increases the risk of chronic diseases such as type 2 diabetes [3] and cardiovascular disease [4]. Exposure to adverse socioeconomic conditions has been shown to be associated with chronic stress [5, 6]. Chronic stress is therefore likely to be one of the explanatory factors for the higher occurrence of adverse health outcomes among people with low socioeconomic status, who are more frequently exposed to such conditions, independent of health-related behaviour [7]. In epidemiological research, socioeconomic status is usually defined based on education, income and/or occupation [8].

One way to *explain* why exposure to adverse socioeconomic conditions is associated with a higher prevalence of chronic stress is to look at the conditions that are necessary for a stress response to arise: high demands and limited access to resources to cope with these demands. The stress response can be conceptualised as being determined by the balance between the demands of a stressor and the resources available to cope with these demands [1, 9]. Specifically, a stress response is more likely to occur when a stressor is assessed as being uncontrollable, i.e. when it poses demands that exceed available resources [10–13]. People exposed to adverse socioeconomic conditions are worse off on both sides: they are likely to endure high demands *and* to have limited access to resources. First, exposure to stressors that pose high demands, such as financial hardship, family conflict, death of a loved one, poor living conditions, crime, violence and (status) discrimination, is correlated with adverse socioeconomic conditions [6, 14]. Second, exposure to adverse socioeconomic conditions implies having limited access to resources, such as money, knowledge, power, prestige and beneficial social connections, to accommodate these demands [15]. In addition to the severity of these stressors as expressed in demands versus resources, exposure to adverse socioeconomic conditions is associated with *recurrent* stressor exposure [16], increasing the probability of suffering from chronic stress.

Epidemiological studies concerning the effect of stressors on health originally focused on finding a linear relationship between stressors—initially expressed as the number of “life events” ranked in severity according to “life change units”—and various outcome measures including distress (i.e. emotional suffering as a function of stressor exposure [17]) [18]. However, it was soon realised that other psychological and social factors are important in defining the impact of these stressors. In fact, across studies it was found that life events only explain anywhere between 1 and 12% of the variance in distress [18]. While availability of resources to accommodate a stressor’s demands are increasingly being taken into account to explain whether or not a stressor evokes a stress response, as described above, it can be argued that looking at the balance between demands and resources is still too simplistic to explain why those exposed to adverse socioeconomic conditions more often suffer from chronic stress. Various reasons can be distinguished that make the relationship between exposure to adverse socioeconomic conditions and chronic stress a complex issue.

First, there are feedback loops between stressor exposure and psychological processes, which results in complexity. This can be illustrated by the reciprocal interaction between stressor exposure and the appraisal of additional stimuli. Recurrent exposure to stressors can lead to an accumulation of stressors, finally resulting in a constant background of chronic stress [2]. This persisting pressure may lead to additional stimuli being assessed as stressors because they are more threatening given the circumstances of chronic stress [1, 11], which results in an amplifying feedback loop.

Second, such interactions are transpiring over different temporal scales. For instance, whether or not a stressor is appraised as controllable is indirectly influenced by past stressor exposure. Here, past experiences of low control can diminish perceived controllability of subsequent stressors [19]. In turn, the effect that past stressor exposure has on perceived controllability is affected by concurrent access to socioeconomic resources [20, 21].

Third, there is an interplay between biological, psychological and social processes, i.e. different spatial scales. For example, because cognitive bandwidth (which relates to the working memory) is a finite resource and each stressor takes up cognitive bandwidth, an accumulation of stressors [6, 14]) can prevent people from addressing some of the stressors [22–25]. The impossibility of dealing with them all at once and the trade-off that therefore needs to be made can leave some of the stressors unresolved [26, 27].

The dynamics described above, stemming from feedback loops and the interconnectedness between temporal and spatial scales, could explain why the relationship between stressors and distress is not linear and why it is necessary to consider biological, psychological and social factors interacting with each other to put this relationship into context [18]. In this paper, we therefore examined the dynamics underlying the relationship between exposure to adverse socioeconomic conditions and chronic stress from the perspective of complexity science, which focuses on dynamics within a system, i.e. on how all parts interact within the whole as opposed to on how each part relates to the whole in isolation [28].

Correspondingly, the research question of this paper was: which dynamics drive chronic stress in a context of adverse socioeconomic conditions? We applied the complexity science perspective to our research question by developing a causal loop diagram (CLD) of the involved dynamics. From this perspective, we examined the impact of a context of adverse socioeconomic conditions on individual-level biological and psychological processes, leading to a biopsychosocial model of stress in line with Lazarus’ model of stress as a relational outcome between people and their environment [11]. Specifically, given that every biopsychosocial model describes an outcome as arising from a combination of the biological, psychological and socio-environmental scales [29], the added value of a complexity science perspective is that it provides methods to explicitly study the interactions between these spatial scales over the life course.

## Methods

In a CLD, empirical research and theoretical knowledge (i.e. well-reasoned hypotheses) can be combined to compose a network of variables and causal links [30]. A CLD is thus a visual representation of “initial hypotheses, or sets of linked hypotheses, about the processes involved” [31]. It is a conceptual framework that can help us interpret the current evidence base.

A CLD is an appropriate methodology to apply the complexity science perspective to our research question for several reasons. First, dynamics can be portrayed in a way that captures important individual differences while retaining a structure that provides an understanding of variation at the population level [32]. Specifically, each causal link refers to a causal mechanism that may act as a driver of chronic stress in a context of adverse socioeconomic conditions. Depending on the exact combination, the dominant mechanisms leading to chronic stress may differ between people exposed to such conditions. Second, a CLD can reveal non-linearity between stressor exposure and chronic stress: it could for instance show where differences in initial conditions may induce large and unforeseeable differences in outcomes through amplifying feedback loops involving biological, psychological and social processes [28]. Third, a CLD allows for interactions between multiple spatial and temporal scales [32].

We conducted a literature review on the mechanisms underlying the relationship between exposure to adverse socioeconomic conditions and chronic stress from a complexity science perspective to develop the CLD, where we focused on the characteristic indicative of a complex system referred to as emergence [31]. Emergent properties “arise from interactions between parts of a system” and “are not seen in any one part of a complex system nor are they summations of individual parts” [31]. This was dictated by our research question addressing the dynamics that drive chronic stress, which may be classified as an emergent property, in a context of adverse socioeconomic conditions. We characterised the stress response—the repeated occurrence of which constitutes chronic stress [2]—as emerging from interactions within a biopsychosocial system [11].

### Procedure

We developed the CLD through “a combination of literature reviews, stakeholder input and discussions within the review team” [31]. This procedure is described below in six steps.

#### Step 1. Drafting the initial version of the causal loop diagram

The modelling group, which consisted of researchers with expertise in public health, health inequalities and complexity science (LC, MN, KS), drafted an initial version of the CLD, including variables and causal links, via group model building (GMB). GMB refers to a participatory modelling technique to explicate the mental models of domain experts concerning a particular system [33]. A CLD that results from GMB represents the consensus of that group regarding how a system works.

#### Step 2. Using literature to substantiate the causal links

The modelling group scoped an extensive body of literature to (1) find evidence either confirming or contesting their perception of this system as exemplified in the initial version; (2) decide on what parts of the system were required to answer the research question (i.e. to define boundaries); and (3) refine the conceptualisation of the variables and causal links proposed in the initial version. Reviews that synthesised empirical evidence and theoretically contextualised this evidence were fundamental to this process [1, 11, 19, 20, 27, 34]. For example, we drew from a review describing “a common conceptual model that incorporates epidemiological, affective and psychophysiological perspectives” for stress [1]. Single studies were used to elucidate specific variables and causal links in the overarching conceptual model. The literature that forms the basis for a particular causal link is included in the text where the causal link is first introduced as well as in Additional file 1: Table S1 [1, 2, 11–13, 19–27, 34–53] for reproducibility and extensibility.

#### Step 3. Defining the boundaries

After scoping the literature, the modelling group defined the boundaries of the system of interest, so as to make the system small enough to comprehend but large enough to cover all aspects relevant to the research question [31, 54]. The boundaries were chosen such that the CLD could explain the consequences of ongoing stressor exposure superimposed on past stressor exposure in adults currently exposed to adverse socioeconomic conditions. Accordingly, the values for past stressor exposure and current socioeconomic resources of a person are used as the initial conditions for describing that person’s dynamics based on the CLD. Adult age was specified so as to integrate the life course perspective (i.e. whether childhood—and to some extent life course—stressors have or have not occurred has already been defined in adult age) and the stress response in one model [1, 5, 18]. To keep the scope of the CLD tailored to the research question (relating to the effect of chronic stress independent of health-related behaviour), health-related behavioural factors in (or as arising from) the stress response, e.g. sleep disruption, enhanced appetite or substance use [55, 56], were not included.

#### Step 4. Using scoped literature to refine the initial version of the causal loop diagram

Within these boundaries, the modelling group iteratively refined the CLD as formulated by one of the researchers (LC) based on the group’s collective understanding of the scoped literature. At several time points during its development, the CLD was presented to an expert in complex systems modelling (RQ).

Firstly, the literature was used to refine the conceptualisation of the variables and causal links. For the variables, this mostly entailed (1) their level of detail; and (2) their specificity. For example, the variable “financial resources” was replaced by “socioeconomic resources” so as to also include knowledge, power, prestige and beneficial social connections [15] (resulting in a lower level of detail) and for the variables involving stressors, i.e. “stressor” and “childhood and life course stressors”, their characteristics, i.e. uncontrollability (“uncontrollable childhood and life course stressors”) and solvability (“solvability of stressors”), were incorporated (resulting in more specificity).

Secondly, for each causal link, the group considered whether mediators should be included to further explicate the mechanisms. For example, while it was already hypothesised that the characteristics of a stressor inform which coping strategy is evoked, this mechanism was further explicated when it was found that uncontrollability of a stressor is an important informing factor (see link 17).

Thirdly, to preserve the CLD’s readability [57], variables were combined into one node where possible. The premises were that each variable collapsed into that node should (1) be modified in a similar manner by incoming causal links; and (2) equivalently influence all variables connected to that node through the outgoing causal links. For example, the node “coping resources” replaced the variables “mastery/self-esteem/locus of control/fatalism”, “neuroticism”, “optimism” and “self-esteem/self-confidence”, as the role of coping resources within the CLD covered all these aspects.

#### Step 5. Identifying feedback loops

At this point, a network of variables and causal links had been established. The modelling group then analysed the CLD in order to identify feedback loops [57]. Feedback loops were identified by taking one of the variables as the starting variable and following the cascade of consecutive causal links pointing in the same direction until either (1) the starting variable was reached again via this cascade or (2) a causal link pointing in the opposite direction was encountered. In the former case, the entire cascade of consecutive causal links beginning and ending with the starting variable was marked as a feedback loop. In the latter case, this particular cascade did not result in a feedback loop and could be disregarded as such. This process was worked through for each variable, successively taking each variable as the starting variable, and for each cascade that could be followed from a particular starting variable. Step 5 was undertaken for each iteration in step 4 in order to make sure that all feedback loops within the network of variables and causal links were identified and saturation was reached. Accordingly, the feedback loops were also iteratively refined.

#### Step 6. Evaluating by asking domain experts for their critical reflection

The CLD was evaluated by presenting it and its written description (including feedback loops) to eight researchers with expertise in social epidemiology, medical demography, psychology, complexity science, mental health, the socioeconomic and psychosocial determinants of health and cognitive and emotional functioning and the long-term effects of childhood adversities on mental health. They were asked to critically reflect on the CLD and to test it against their knowledge, from their specific domain, of the subject. The feedback of each of the domain experts consulted was found to be largely overlapping, which was taken as an indication of consensus and saturation, and was discussed in the modelling group together with one of the contributing researchers with expertise in psychology and complexity science (AS). Final adaptations were then made, primarily relating to refining the terminology used, so as to be unambiguous between disciplines, and to contextualising the CLD to exposure to adverse socioeconomic conditions within the written description.

### Reading the causal loop diagram

The definitions of the concepts used in either the CLD or its description are given in Table 1.

**Table 1:** Definitions of concepts in alphabetical order

Variable	Definition	
Chronic stress	The repeated occurrence of the stress response over an extended period of time, i.e. several hours per day during weeks to months [2].	
Cognitive bandwidth	Relates to the working memory, includes cognitive capacity, i.e. those psychological processes that govern capability to “solve problems, retain information, engage in logical reasoning, and so on”, and executive control, i.e. those psychological processes that govern capability to “manage [ … ] cognitive activities, including planning, attention, and initiating and inhibiting actions” [23, 25].	
Controllability	A person’s assessment of whether their behavioural responses could considerably alter the outcomes of the stressor [1, 58]. Controllability therefore only exists relative to a person.	
Coping resources	Those immaterial individual differences that can increase capability to manage stressors, which can be seen as “antecedents of specific coping strategies” [34], affecting which coping strategy is invoked under stressor exposure.	
	**Mastery/self-efficacy/locus of control/fatalism**	Are intertwined with control: each pertains to the self-perceived influence on own behaviour, the situation and the future [59, 60]. There are subtle differences: mastery, locus of control and fatalism are descriptive of a general sense of control, whereas self-efficacy is often used to refer to a sense of control in a specific situation [59].
	**Neuroticism**	Often referred to as a character trait resulting from inter-individual variation in “negative emotional response to threat, frustration, or loss” and typified by “irritability, anger, sadness, anxiety, worry, hostility, self-consciousness, and vulnerability” [47]. It can, in contrast, also be defined as a tendency to be subjected to strong negative emotions that are paired with an assessment of uncontrollability when faced with a stressor [36].
	**Optimism**	Anticipation of positive future events [42].
	**Self-esteem/self-confidence**	Are closely related, where self-esteem is a global trait belief, i.e. “an attribute of a person”, and self-confidence is a specific state belief, i.e. “an attribute of a person-in-a-situation” [61, 62].
Distress	Emotional suffering as a function of stressor exposure [17].	
Primary appraisal	Describes the process during which it is established whether a stimulus is assessed as a threat or a challenge (precedes secondary appraisal) [11, 63].	
Problem avoidance (relative to problem approaching)	Problem avoidance refers to disengaging from a stressor, while problem approaching refers to trying to understand, find a solution to and accept a stressor [27, 64]. Strategies associated with problem avoidance are negatively correlated with strategies associated with problem approaching, implying that more problem avoidance begets less problem approaching and vice versa [26, 27]. Note that problem avoidance is often contrasted with problem solving instead of problem approaching, where problem solving can be referred to as “active cognitive and behavioural efforts to deal with the problem” [27]. Even though problem solving is often used with the same implications as problem approaching in this context, we used problem approaching as the opposite of problem avoidance because if a person is not avoiding a problem it does not necessarily mean that they are able to solve it. We used the terms problem avoidance and problem approaching to cover both behavioural and emotional aspects of coping, where both aspects appeared to be influenced by the included causal links in the same way. For example, problem avoidance can cover both behavioural disengagement, e.g. not undertaking efforts to manage a stressor, and/or emotional disengagement, e.g. suppressing the distress associated with a stressor. In the CLD the variable reflective of a person’s coping strategy is referred to as "problem avoidance *relative to problem approaching*" because in reality people are likely to mix problem avoidance and problem approaching.	
Reappraisal	Re-evaluating the meaning of a stimulus over a period of time [65].	
Secondary appraisal	Describes the process of assessing whether the resources available can meet the demands raised by a stressor, which determines whether the stressor is appraised as controllable or uncontrollable (follows primary appraisal) [11–13].	
Socioeconomic resources	Include “money, knowledge, power, prestige, and the kinds of interpersonal resources embodied in the concepts of social support and social network” [15].	
Solvability of stressors	Refers to what people can *actually* do to alter the outcomes of stressors.	
Stimulus	Refers to “any agent, event, or situation—internal or external—that elicits a response” [66].	
Stress response	Those cognitive, affective and biological reactions induced by a stressor [1].	
Stressor	A stimulus which is appraised as stressful, having been identified as a threat as opposed to a challenge [11, 63].	

A distinction was made between exogenous, i.e. non-feedback, and endogenous, i.e. feedback, variables in the CLD’s description, where the former represent initial conditions driving the dynamics and the latter are involved in reciprocal relationships indicated by feedback loops.

**Fig. 1:**
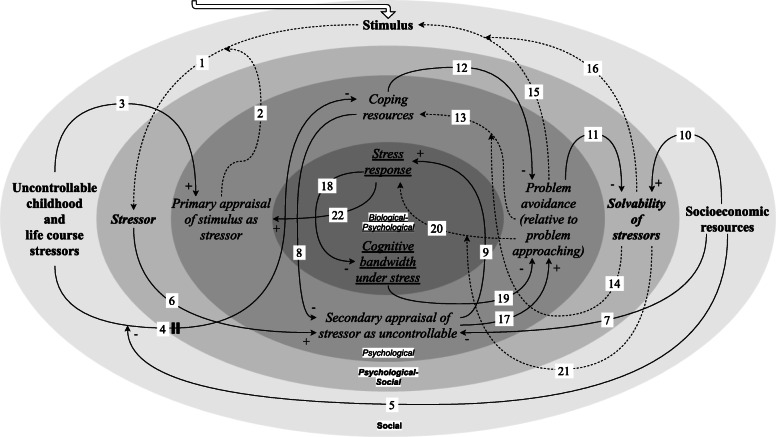
Causal loop diagram. Positive (+; an *increase* in the first variable causes an *increase* the second) and negative (−; an *increase* in the first variable causes a *decrease* in the second) causal links are marked accordingly. Changeable causal links (i.e. without a fixed positive or negative polarity) are depicted as dashed arrows, where the polarity or strength of the effect is dependent on the variable at the tail of the intercepting changeable causal link (link 1/2, 4/5, 13/14, 15/16, 20/21). The perpendicular double line on link 4 signifies that this causal link acts on a considerably different temporal scale than the other causal links. The biological, psychological and social spatial scales are visualised as underlined, in italics and in bold, respectively, and are depicted as different layers indicated by various shades of grey. Combinations are also demonstrated. We included the variable “stimulus” in the social spatial scale because the nature of the stimuli that people are exposed to is intertwined with exposure to adverse socioeconomic conditions [6, 14]

**Fig. 2:**
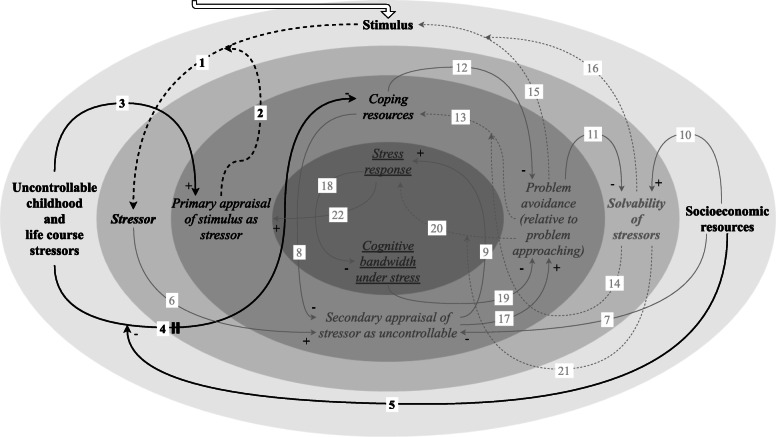
Initial condition: uncontrollable childhood and life course stressors. Exposure to adverse socioeconomic conditions increases the likelihood of exposure to uncontrollable childhood and life course stressors. Past exposure to such stressors is likely to influence primary appraisal for subsequent stimuli and access to coping resources

**Fig. 3:**
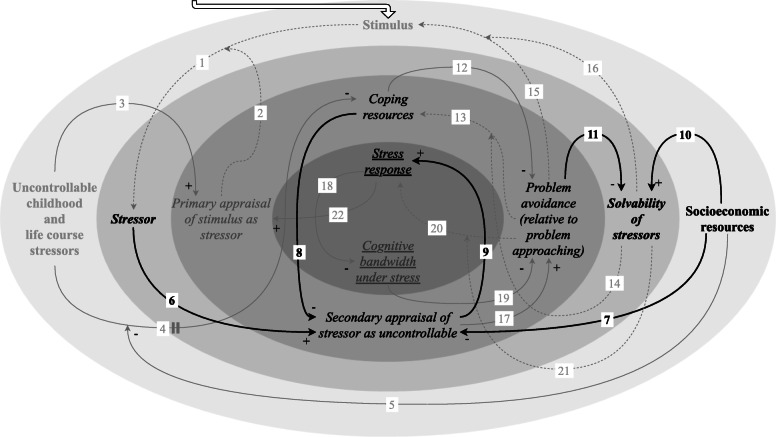
Initial condition: socioeconomic resources. Socioeconomic resources influence secondary appraisal of stressors as well as their solvability

**Fig. 4:**
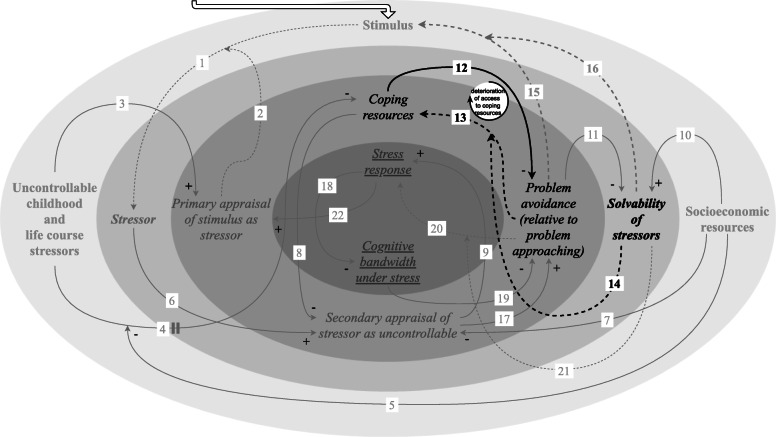
Feedback loop: progressive deterioration of access to coping resources because of repeated insolvability of stressors. This feedback loop underlies the progressive deterioration of access to coping resources because of the repeated insolvability of stressors. Exposure to adverse socioeconomic conditions is associated with being repeatedly confronted with insolvable stressors. If problem approaching is evoked when a stressor is insolvable, this can undermine access to coping resources. Undermined access to coping resources can in turn evoke problem avoidance, which can progressively deteriorate coping resources if stressors are solvable

**Fig. 5:**
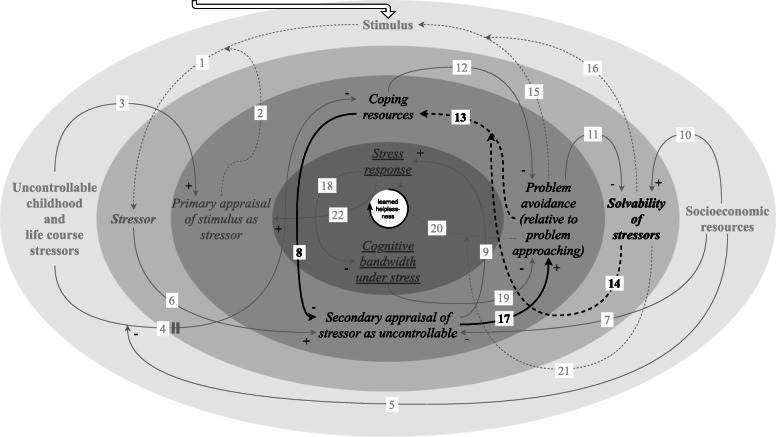
Feedback loop: perception of stressors as uncontrollable due to learned helplessness. This feedback loop explains how the secondary appraisal of a stressor as uncontrollable may originate from undermined access to coping resources. Past exposure to uncontrollable stressors, which is associated with adverse socioeconomic conditions, can undermine access to coping resources. The secondary appraisal of a stressor as uncontrollable may evoke problem avoidance, which may in turn further undermine access to coping resources, making it even more likely that stressors are appraised as uncontrollable. In the literature, the phenomenon emerging from this amplifying feedback loop has been described as learned helplessness

**Fig. 6:**
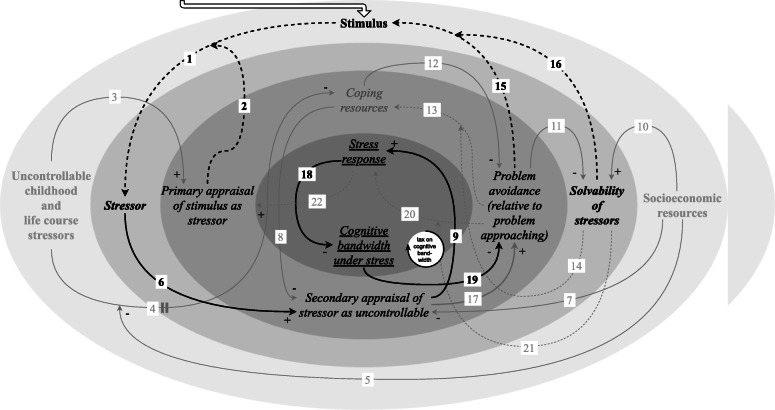
Feedback loop: tax on cognitive bandwidth caused by the stress response and chronic stress. This feedback loop demonstrates the reciprocal relationship between stressor exposure, which people exposed to adverse socioeconomic conditions are recurrently subjected to, and cognitive bandwidth under stress. As cognitive bandwidth is a finite resource and each stressor takes up cognitive bandwidth, an accumulation of stressors can prevent people from addressing some of the stressors. The impossibility to deal with them all at once and the trade-off that therefore needs to be made can leave some of the stressors unresolved, which then keep taking up cognitive bandwidth

**Fig. 7:**
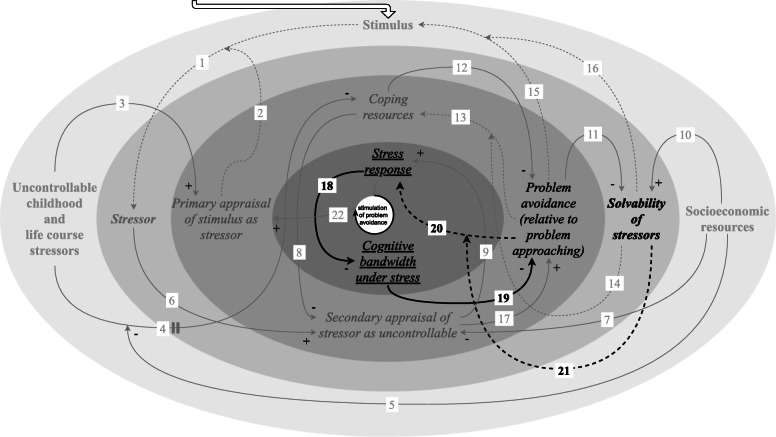
Feedback loop: stimulation of problem avoidance to provide relief from the stress response and free up cognitive bandwidth. Exposure to adverse socioeconomic conditions is correlated with recurrent stressor exposure. An accumulation of insolvable stressors can stimulate problem avoidance, in order to provide relief from the stress response and concurrently free up cognitive bandwidth

**Fig. 8:**
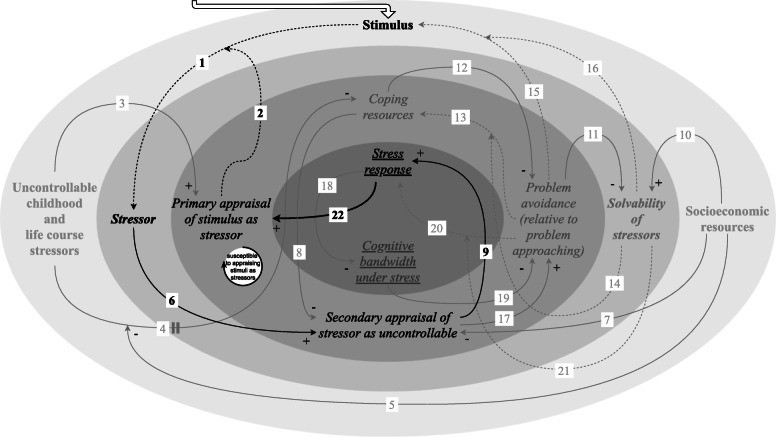
Feedback loop: susceptibility to appraising stimuli as stressors against a background of chronic stress. This feedback loop describes that when people are enduring chronic stress, which is associated with exposure to adverse socioeconomic conditions, they may be more susceptible to appraising additional stimuli as stressors

**Fig. 9:**
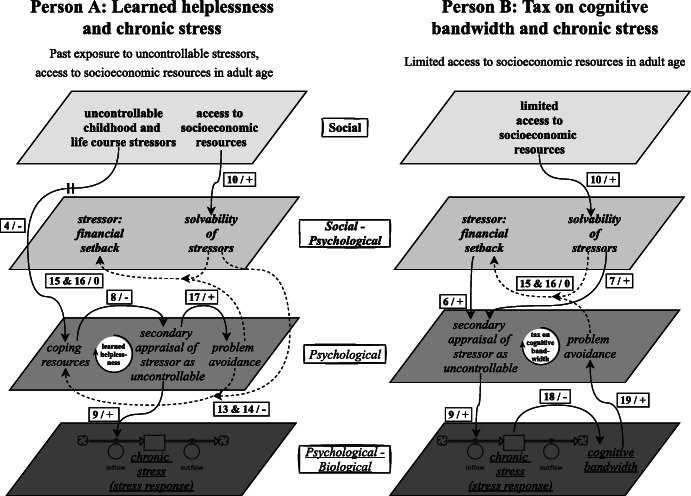
Two scenarios showing how exposure to adverse socioeconomic conditions can affect people in different ways and that the dominant mechanisms leading to chronic stress may vary between people within such a context. The scenarios are representative of two of the CLD’s feedback loops, relating to learned helplessness (Fig. 3; section “Perception of stressors as uncontrollable due to learned helplessness”) and tax on cognitive bandwidth (Fig. 4; section “Tax on cognitive bandwidth caused by the stress response and chronic stress”). Chronic stress is depicted as a stock, which is according to the representation of a system’s dynamics as a stock-and-flow diagram [54]. A stock indicates “an accumulation of material or information that has built up over time” [54], and thus can illustrate the repetition of the stress response constituting chronic stress. A stock is conventionally shown as a box with an inflow and an outflow, which represent information or material flowing into or out of the stock [54]. For instance, in accordance with Figs. 1, 2, 3, 4, 5, 6, 7 and 8, secondary appraisal of a stressor as uncontrollable evokes a stress response, which contributes to the build-up of chronic stress (link 9). Variables are depicted across four spatial scales—variables that overlap between the social and psychological scales and between the psychological and biological scales are represented accordingly. Each link is marked with a number and its polarity, corresponding to its depiction in Figs. 1, 2, 3, 4, 5, 6, 7 and 8. The effect of the changeable causal links is represented for these specific scenarios (see also Additional file 1: Table S1 for the polarity of the changeable causal links under various conditions). For instance, the combination of link 15/16 has the same effect (i.e. no change) in each of the scenarios, but for different reasons. The link marked with a perpendicular double line is subjected to a delay (person A)

In a CLD, causal links between variables are visualised by directed arrows, usually with polarities for positive (+) or negative (−) causal links, where a positive causal link indicates that an *increase* in the variable at the tail of the arrow constitutes an *increase* in the variable at the head of the arrow, whereas a negative causal link indicates that an *increase* in the variable at the tail constitutes a *decrease* in the variable at the head. Various causal links—as indicated with a tilde (~) in Additional file 1: Table S1 and depicted as dashed arrows in Figs. 1, 2, 3, 4, 5, 6, 7 and 8 —are not accompanied by a fixed positive or negative polarity. This is to allow for individual variation in the dominant mechanisms leading to chronic stress in a context of adverse socioeconomic conditions, as is illustrated in the two hypothetical scenarios introduced in the next section. The combination of a first changeable causal link connecting two variables (from variable *x* to variable *y*) and a second changeable causal link intercepting this first link (from variable *z* to the link from variable *x* to variable *y*) (link 1/2, 4/5,13/14, 15/16, 20/21) is used to signify that the polarity or the strength of the first changeable causal link (from variable *x* to variable *y*) is dependent on the variable at the tail of the second changeable causal link (variable *z*). This means that in some combinations of values for variable *x* and variable *z*, there is *no change* (0) in variable *y*, as illustrated in Additional file 1: Table S1 and Fig. 9. Lastly, the arrow marked with a perpendicular double line is subjected to a delay, where we used this mark to signify that this causal link acts on a considerably different temporal scale than the other causal links (link 4).

As spatial scales, we took biological, psychological and social variables. These spatial scales are visualised as underlined, in italics and in bold, respectively, in the CLD and are depicted as different layers indicated by various shades of grey (Figs. 1, 2, 3, 4, 5, 6, 7 and 8). Combinations are also demonstrated: in the stress response, for instance, both the biological and the psychological are interdependently involved. The temporal scales (not visualised in the CLD, discussed in its description) range from the primary appraisal affecting the stress response, which functions *on an acute basis*; through the interrelationships between exposure to stimuli, which can change *on a daily basis*; to primary appraisal, which can be influenced by chronic stress *on a lifetime basis*.

### Scenarios

To elicit from the resulting CLD how the interaction between the exogenous and endogenous variables could vary between people, leading to different dominant mechanisms resulting in chronic stress in a context of adverse socioeconomic conditions, we formulated two hypothetical scenarios (similar to Kenzie et al. [32]), which we then compared to each other. These scenarios are examples of how variation can emerge from the CLD and are not meant to capture all the ways in which the interaction between variables could vary between people.

## Results

### Causal loop diagram

Each causal link in the CLD (Fig. 1) is described in relation to its role as connecting exogenous (section “Exogenous variables: initial conditions”; Figs. 2 and 3) or endogenous variables (section “Endogenous variables: feedback loops”; Figs. 4, 5, 6, 7, 8). The CLD contains two exogenous variables: “uncontrollable childhood and life course stressors” (section “Uncontrollable childhood and life course stressors”); and “socioeconomic resources” (“Socioeconomic resources”). We use the word “uncontrollable” to refer to a person’s assessment of whether their behavioural responses could considerably alter the outcomes of the stressor [1, 58]. As specified above, these were taken to be the initial conditions for the unfolding mechanisms described. The nine remaining variables are endogenous variables, which are involved in five feedback loops. The variable “stimulus” is categorised as an endogenous variable and not as an initial condition, because it is involved in feedback loops. However, an arrow pointing in the direction of the variable “stimulus” was also added to clarify that this variable is contextual and that stimuli can continue to arise as inputs to the system given the accumulation of stressors that is associated with exposure to adverse socioeconomic conditions. Each feedback loop is labelled for its contribution to explaining the dynamics that drive chronic stress in a context of adverse socioeconomic conditions: the feedback loops correspond to (1) progressive deterioration of access to coping resources because of repeated insolvability of stressors (section “Progressive deterioration of access to coping resources because of repeated insolvability of stressors”); (2) perception of stressors as uncontrollable due to learned helplessness (“Perception of stressors as uncontrollable due to learned helplessness”); (3) tax on cognitive bandwidth caused by the stress response and chronic stress (“Tax on cognitive bandwidth caused by the stress response and chronic stress”); (4) stimulation of problem avoidance to provide relief from the stress response and free up cognitive bandwidth (“Stimulation of problem avoidance to provide relief from the stress response and free up cognitive bandwidth”); and (5) susceptibility to appraising stimuli as stressors against a background of chronic stress (“Susceptibility to appraising stimuli as stressors against a background of chronic stress”).

### Exogenous variables: initial conditions

#### Uncontrollable childhood and life course stressors

During primary appraisal, it is established whether a stimulus is assessed as a threat or as a challenge [11] (Fig. 2). Accordingly, a stressor is a stimulus that is appraised as a threat (link 1/2) [11]. Primary appraisal is in part dependent on past exposure to uncontrollable stressors [1]. It has been reported that exposure to adverse socioeconomic conditions increases the likelihood of exposure to uncontrollable childhood and life course stressors [5, 19, 52].

To understand the effect of past stressor exposure on the primary appraisal of a current stimulus (link 3), the brain has been characterised as a “prediction machine” that derives its primary appraisal not only from the features of the stimulus but also from a person’s “personal memory bank” [1]. This memory bank is marked by previous experiences, where “a history of feeling or being threatened might shift the appraisal of a current stimulus to more of a threat than challenge appraisal” [1].

Exposure to uncontrollable childhood and life course stressors can also undermine access to coping resources in adulthood (link 4) [19, 35–43]. We focused on those four coping resources that we found to be affected by stressor exposure: mastery/self-efficacy/locus of control/fatalism, neuroticism, optimism and self-esteem/self-confidence (definitions provided in Table 1).

Access to each of these coping resources is likely to be negatively affected by uncontrollable childhood and life course stressors [19, 35–43]. This may be related to these experiences leaving less opportunities for the development of coping resources. For example, because a general sense of self-efficacy evolves through specific mastery experiences, adverse mastery experiences—where the situation could not be controlled—can undermine self-efficacy [35]. Self-efficacy thus develops through favourable mastery experiences over the life course, implying that adverse mastery experiences are particularly detrimental when self-efficacy has yet to be developed [35], as is the case for children.

Still, socioeconomic resources in adulthood could facilitate reappraisal of stressors that were previously perceived as uncontrollable (link 5) [20, 21]. Specifically, the extent to which past exposure to such stressors still impacts access to coping resources in adulthood may be modified by socioeconomic resources. Socioeconomic resources may influence whether some level of resolution has been reached as more resources became available over time, even if the stimulus was perceived as an uncontrollable stressor at the moment it occurred. To support this, an analysis of life course pathways leading from adverse childhood experiences towards adult psychological well-being—including distress—showed that social support, as measured by a sense of community, in adulthood can buffer the effect of adverse childhood events on perceived well-being in adulthood [21].

#### Socioeconomic resources

Secondary appraisal entails assessing whether the resources available can meet the demands raised by a stressor (link 6), which determines whether the stressor is appraised as controllable or uncontrollable [11–13] (Fig. 3). In addition to the demands posed by the stressor in question, access to socioeconomic resources (link 7) and coping resources (link 8) also determine the assessment of the stressor’s controllability [11–13, 44]. Exposure to adverse socioeconomic conditions is correlated with exposure to stressors with high demands [6, 14]. Moreover, people exposed to such conditions are more likely to have limited access to socioeconomic resources to accommodate these demands [15] and to have been previously exposed to uncontrollable stressors that have undermined their access to coping resources, as detailed above. This combination makes it more likely that a stressor is assessed as uncontrollable—instigating a stress response (link 9) [11]. Link 6, 7 and 8 together illustrate the balance between exogenous variables (demands posed by a stressor (stemming from a stimulus) (link 6) and access to socioeconomic resources to accommodate those demands (link 7)) and endogenous variables (access to coping resources (link 8)) in determining how a stressor is assessed. For example, whether long working hours result in a stress response may depend on a person’s evaluation of their autonomy in their work environment, dependent on their position within the organisation [44]. Chronic stress can develop via link 9 through the chronicity of exposure to stressors assessed as uncontrollable and correspondingly the repetition of the stress response [2]. As additional stimuli are likely to keep arising over time in a context of adverse socioeconomic conditions, this can result in chronic stress.

Limited access to socioeconomic resources decreases the solvability of stressors (link 10). Solvability of stressors refers to what people can *actually* do to alter their outcomes and is influenced by access to socioeconomic resources and by possible coping strategies. Firstly, having access to socioeconomic resources increases what is within people’s power to change the outcomes of stressors, increasing the solvability of stressors (link 10). Secondly, the solvability of stressors is affected by which coping strategies are possible given the circumstances (link 11), as is further explained in section “Progressive deterioration of access to coping resources because of repeated insolvability of stressors”.

### Endogenous variables: feedback loops

#### Progressive deterioration of access to coping resources because of repeated insolvability of stressors

We described above that past exposure to uncontrollable stressors, associated with adverse socioeconomic conditions, can undermine access to coping resources [19, 35–43] (Fig. 4). Exposure to adverse socioeconomic conditions increases the likelihood of being repeatedly confronted with insolvable stressors. If problem approaching is evoked when a stressor is insolvable, this can undermine access to coping resources as well (link 14) [19, 27, 52]. For example, actively engaging in situations that children are not able to change, such as inter-parental conflict, can result in worse outcomes for them on a behavioural and emotional level, which teaches them not to attempt this strategy when faced with subsequent stressors [19]. This can leave access to coping resources undermined, while access to coping resources affects coping strategies. Undermined access to each of the included coping resources can evoke problem avoidance, while problem approaching may be a consequence of access to the included coping resources (link 12) [27, 45–49].

This mechanism is part of an amplifying feedback loop, as undermined access to coping resources can prevent problem approaching for solvable stressors—a combination which can result in a further undermining of access to coping resources (link 13/14) [19, 20, 27, 36, 50–52]. If stressors are solvable, the coping strategy that is induced by undermined access to coping resources, i.e. problem avoidance, can further restrict this access, whereas the coping strategy allowed for by access to coping resources, i.e. problem approaching, can further increase this access (link 14) [19, 27, 52]. A coping strategy thus either increases or decreases access to coping resources, possibly creating a virtuous cycle for those who already have access to coping resources and a vicious cycle for those whose access to coping resources has been undermined in the past. For example, optimism is related to “an active approach mode”, i.e. problem approaching, which facilitates the feeling of accomplishment after a favourable coping experience and in turn reinforces optimism regarding the future (“an ‘I can do it’ attitude”) [51]. Conversely, Aldwin et al. suggest that people who are low in mastery at baseline may have no opportunities for problem approaching, which may lead to reduced “situation-specific mastery”, which in turn may result in even lower “global mastery” as compared to the baseline [20]. In turn, once access to coping resources has been undermined, problem avoidance coping strategies are more likely to be invoked, which can successively further restrict access to coping resources—creating an amplifying feedback loop.

If stressors are insolvable, coping strategies associated with disengagement are actually correlated with lower stress (link 14) [19, 27, 52]. Here, “the impracticality and even the danger of more ‘active’ problem-solving techniques” that more often than not are evident in a context of adverse socioeconomic conditions should be acknowledged [52].

#### Perception of stressors as uncontrollable due to learned helplessness

Access to coping resources of people exposed to adverse socioeconomic conditions has more often been undermined by past exposure to uncontrollable stressors, which increases the likelihood of appraising subsequent stressors as uncontrollable (link 8) [11–13, 44] (Fig. 5). The secondary appraisal of a stressor as uncontrollable informs which coping strategy is adopted. In particular, if a stressor is assessed as being uncontrollable, it makes less sense to employ a problem approaching coping strategy than if the stressor is assessed as controllable—because an uncontrollable stressor will probably persist regardless of the coping strategy [2]. Accordingly, if a stressor is assessed as uncontrollable, problem avoidance can be the least harmful strategy (link 17) [2, 53]. Despite “active, thoughtful, and creative strategies to cope with the difficult problems” in the face of limited access to socioeconomic resources, problem approaching coping strategies often do not meet their objective due to the circumstances [52].

We explained that if problem approaching is evoked when a stressor is insolvable, this can undermine access to coping resources over time, which Belle and Doucet describe as “repeated coping failures may then lead to the belief that stress factors cannot be overcome” [52] (link 13/14) [19, 20, 27, 36, 50–52]. As access to coping resources progressively gets more limited, because of the lack of options available for problem approaching, the probability that stressors are assessed as uncontrollable increases. This amplifying feedback loop may gradually result in an increasing number of stressors being appraised as uncontrollable and, consequently, in an overwhelming accumulation of stressors that may have not been assessed as threats in the past but have become threats given the circumstances, making it impossible for all stressors to be addressed.

Low actual control, defined in the literature as low “control that individuals are able to exercise over their living environment through the economic and socioeconomic resources they have at their disposal” [67], and low perceived control, defined as low control beliefs that can arise from socialisation in environments characterised by adverse socioeconomic conditions, are often separated in explanatory models concerning health inequalities [67]. The relationship between a context of adverse socioeconomic conditions and chronic stress is represented as a linear process, commencing either with what people can *actually* do or with what they *think* they can do (as learned by others) to alter the outcomes of a stressor. Looking at this relationship as an amplifying feedback loop can consolidate these two pathways, showing that actual and perceived control are interlinked, where low perceived control does not necessarily originate from socialisation, but can also be empirically deduced from past experiences. It is rational to have “the belief that stress factors cannot be overcome” [52] if all past experiences under the same circumstances substantiate this belief. This mechanism is indicated in this amplifying feedback loop: low perceived control may stem from undermined access to coping resources, which in turn may be caused by past exposure to uncontrollable stressors and the repeated insolvability of stressors. This suggests that low *perceived control* can be the result of past experiences of low *actual control*. The phenomenon emerging from this amplifying feedback loop has been referred to as learned helplessness [53].

#### Tax on cognitive bandwidth caused by the stress response and chronic stress

Exposure to adverse socioeconomic conditions is correlated with recurrent exposure to stressors [16], which can result in the repeated occurrence of the stress response (link 9) [11], i.e. chronic stress [2] (Fig. 6). The stress response in turn is accompanied by another amplifying feedback loop. A stress response taxes working memory, leaving less cognitive bandwidth for other tasks (link 18) [22–25]. Cognitive bandwidth has extensively been researched against the background of scarcity, which is a prominent stressor associated with exposure to adverse socioeconomic conditions. It has been shown that the “internal disruptions” and “involuntary preoccupation” associated with scarcity affect cognitive capacity (i.e. those psychological processes that govern capability to “solve problems, retain information, engage in logical reasoning, and so on”) and executive control (i.e. those psychological processes that govern capability to “manage [ … ] cognitive activities, including planning, attention, and initiating and inhibiting actions”) [23, 25].

An accumulation of stressors, against the background of exposure to adverse socioeconomic conditions [6, 14], can be detrimental because cognitive bandwidth is a finite resource and each stressor takes up cognitive bandwidth [22–25]. It is important to note that cognitive bandwidth is thus dependent on background processes, but independent of inherent traits [22] (hence the term “bandwidth”, which in computing refers to “the amount of information that can be sent over a network connection at one time” [68]). These changes in cognitive capacity and executive control as a function of the stress response can be traced back to the biological underpinnings of the stress response [24].

A tax on cognitive bandwidth is one of the explanatory factors for why an accumulation of stressors can prevent people from addressing some of the stressors, given the impossibility to deal with them all at once and the trade-off that therefore needs to be made (link 19) [22–25]. This can result in an amplifying feedback loop where it is impossible to accommodate the demands of current and/or additional stressors due to the fact that they are accumulating, leaving some of the stressors unresolved (link 15/16) [26, 27], which then continue to take up cognitive bandwidth.

#### Stimulation of problem avoidance to provide relief from the stress response and free up cognitive bandwidth

Exposure to adverse socioeconomic conditions is correlated with recurrent stressor exposure [16] (Fig. 7). As outlined above, an accumulation of stressors can sustain multiple amplifying feedback loops, presumably operating simultaneously, including a tax on cognitive bandwidth (link 19) [22–25] resulting from the repeated occurrence of the stress response (link 18) [22–25].

As has been noted previously by others, problem avoidance is often wrongly presented as a coping strategy that is maladaptive in all circumstances [69]. Specifically, previous empirical research indicates that coping strategies associated with problem avoidance are adaptive for stressors that are temporary and uncontrollable [34]. Analogously, problem approaching is accommodating when a stressor is solvable, but may be maladaptive and even lead to adverse outcomes when it is not [27]. The “impracticality and even the danger of more ‘active’ problem-solving techniques” [52] in the face of an accumulation of insolvable stressors should be taken into account to recognise that problem avoidance can be the least harmful strategy in a context of adverse socioeconomic conditions in order to provide relief from the stress response (link 20/21) [27, 34], and make cognitive bandwidth available for subsequent stressors that might be solvable.

#### Susceptibility to appraising stimuli as stressors against a background of chronic stress

People exposed to adverse socioeconomic conditions are more likely to be faced with recurrent stressor exposure [16] (Fig. 8). If the stress response keeps manifesting, i.e. becomes chronic stress [2], this may result in changes in primary appraisal of additional stimuli. Particularly, an additional stimulus may be more likely to be perceived as a threat (link 22) [1].

If the stress response is “sustained or slow to return to baseline”, it is associated with “prolonged anticipation of future events, elevation of affective states such as anxiety and worry, or physiological states of vigilant preparedness, reflected in autonomic nervous system arousal” [1]. While people are experiencing chronic stress driven by a context of adverse socioeconomic conditions, subsequent stress responses might thus be different, where additional stimuli may be more likely to be evaluated as threats. It is however uncertain whether a background of chronic stress always results in additional stimuli being more likely to be appraised as threats. Chronicity of the stress response may, over time, also result in a dampening of the stress response to additional stimuli [1]. Nevertheless, it appears that, at least in some cases, this amplifying feedback loop contributes to chronic stress.

### Scenarios

We compared and contrasted two hypothetical scenarios, relating to person A and B, as depicted in Fig. 9. These scenarios are illustrative of how exposure to adverse socioeconomic conditions can affect people in different ways and that the dominant mechanisms leading to chronic stress may differ between people within such a context. Specifically, the CLD, while the structure of the variables and the causal links remains the same, can account for individual variation in two ways. Firstly, the values for the variables relating to a specific person, such as the extent to which they have been exposed to uncontrollable stressors in the past and the precise combination of socioeconomic resources that they have access to, can cause individual variation. Secondly, the strength of the effect of a causal link may differ between people, where for example the negative effect of past exposure to uncontrollable stressors on current access to coping resources may not be equally strong among different people.

In these two scenarios, person A and B are dissimilar in their initial conditions: A was exposed to adverse socioeconomic conditions during childhood and has been exposed to uncontrollable stressors, whereas B is currently exposed to adverse socioeconomic conditions. A has access to socioeconomic resources in adult age, while B has limited access (link 10). Both A and B are affected by a financial setback. They each appraise this stressor as uncontrollable, leading to a stress response (link 9). The cause for this secondary appraisal however differs between A and B—A’s access to coping resources has been undermined by past stressor exposure (link 4, link 8), while B is unable to resolve the stressor because of a context of adverse socioeconomic conditions (link 6, link 7). Both have no options for problem approaching, via different mechanisms: for person A this is because of learned helplessness (link 17, link 13/14), where their “belief that stress factors cannot be overcome” [52] is grounded in past experiences, whereas person B’s cognitive bandwidth is continually taxed by the financial setback because they do not have the socioeconomic resources to accommodate the demands of this stressor (link 18, link 19). The persistence of the stress response induced by the financial setback causes chronic stress as, given a context of adverse socioeconomic conditions, the stressor is insolvable for both person A and person B (link 15/16).

## Discussion

In this paper, we aimed to identify the dynamics that drive chronic stress in a context of adverse socioeconomic conditions. We started the introduction with the conceptualisation of the stress response as being determined by the balance between the demands of a stressor and the resources available to accommodate these demands. By taking a complexity science perspective, we have identified multiple amplifying feedback loops which may be elicited following exposure to a context of adverse socioeconomic conditions, showing how these demands and resources are interlinked in a complex way. For example, stressor exposure affects psychological processes that are important in accommodating subsequent stressors, which can result in a non-linear relationship between stressors and chronic stress. In a context of adverse socioeconomic conditions, it seemed that the impact of stressors on chronic stress increases over time, dictated by the continuous accumulation of stressors and the elimination of options for problem approaching. The fact that a context of adverse socioeconomic conditions comes with a constant exposure to stressors over the life course appeared to be essential in the dynamics identified. The combination of recurrent exposure to stressors and undermined access to coping resources due to past exposure to uncontrollable stressors can inform future assessments of stressors as uncontrollable. Coupled with the experience that stressors could not be overcome in the past, this can lead to feelings of helplessness in relation to current stressors, thereby increasing chronic stress. As a consequence of this chronic stress, cognitive bandwidth is taxed, which can leave stressors unresolved, amplifying chronic stress. In addition, a background of chronic stress can increase the susceptibility to appraising additional stimuli as threats. Because these feedback loops are interlinked, these dynamics could be conceptualised as one vicious cycle. Given the higher probability of recurrent exposure to stressors, this vicious cycle is more likely to occur and to be unceasingly sustained over the life course for people exposed to adverse socioeconomic conditions.

The first reason why we took a complexity science approach was that it enabled us to do justice to the interactions between the involved biological, psychological and social factors that are needed to put the relationship between stressors and distress into context. This perspective can therefore lead to a new outlook on how these factors should be addressed in interventions. The apparently decisive role of these interactions implies that there may be room for improvement in the way we address the role of stress in interventions. The CLD shows that a context of exposure to adverse socioeconomic conditions can mean that problem approaching coping strategies have repeatedly been proven to be unsuccessful because stressors are insolvable, which can result in “the belief that stress factors cannot be overcome” [52] and affect the appraisal of subsequent stressors. This indicates that improving access to socioeconomic resources as a strategy to reduce chronic stress might need to be accompanied by attention to past experiences that may have substantiated feelings of helplessness.

Consequently, we should take care not to conceptualise chronic stress as an individual-level issue but as the emergent outcome of a set of conditions. However, individual-level interventions focusing on such psychological processes have increasingly become established practice in this context over the past years. The pitfall of this type of interventions is that they can draw attention away from the contextual circumstances that cause recurrent exposure to stressors and shift responsibility to the individual. An example of an intervention concentrating on psychological processes is Mobility Mentoring™, which aims to “teach clients how to ‘mentor’ themselves, so that they can better analyse their own problems, regulate their own behaviours, independently set long-term and short-term goals, build mastery and self-control, and transfer those skills to others by mentoring their own children, communities, and networks” [70]. Translated to the CLD, the goal of this intervention is to invoke behaviour change in people regarding coping resources and coping strategies. The CLD illustrates that if the social context remains the same, individual-level psychological processes might not be the most productive place to start to mitigate chronic stress. This is because, as demonstrated by the CLD, psychological processes are affected by exposure to stressors that pose high demands. Despite its focus on psychological processes, Mobility Mentoring™ is advocated as a means to ultimately move out of poverty [71]. In other words, the assumption is that devoting effort to psychological processes should eventually go hand in hand with mobilising socioeconomic resources. The CLD serves to emphasise that even if interventions such as the one described above can realise shifts in psychological processes, these improvements can only be maintained if the accumulation of stressors and their repeated insolvability are also alleviated. The amplifying feedback loops relating to learned helplessness and to deterioration of access to coping resources because of repeated insolvability of stressors both illustrate the necessity of simultaneously targeting contextual circumstances and psychological processes.

Analogously, in the context of the obesity epidemic, public health researchers now agree that obesity is more than an individual-level energy imbalance [72]. It is the final embodied outcome of a constellation of factors ranging from epigenetics and social norms to the built environment and market forces [73]. It seems appropriate to create a similar narrative for chronic stress. Specifically, in the context of obesity it is now realised that “a siloed focus on individual responsibility leads to a failure to address these wider factors for which government policy can and should take a leading role” [73]. Looking at chronic stress as purely a mental health concern could mean erroneously individualising a social issue. Paying undue attention to individual-level psychological processes independent of access to socioeconomic resources could steer in the direction of blaming the victim.

The second reason why we took a complexity science perspective was for its potential to explain non-linearity as arising from amplifying feedback loops. Amplifying feedback loops in a system can cause small differences in initial conditions to be amplified over time, resulting in large differences in the emergent properties of the system. The CLD shows how exposure to uncontrollable stressors can be understood as an initial condition that may steer the system towards a certain outcome, highlighting this condition as a promising leverage point for changing system-level outcomes. In complexity science, leverage points refer to “places in the system where a small change could lead to a large shift in [system-level] behavior” [54]. As differences in initial conditions may start out small but gradually push people towards entirely different trajectories, the timing of interventions within the life course appears crucial. Even though we defined “uncontrollable childhood and life course stressors” as a non-feedback variable in the CLD, in reality this initial condition is not independent of context and will be driven by social structures. Accordingly, the system that we described is evolving within a larger system, which should be considered in order to affect this initial condition, meaning that exposure to adverse socioeconomic conditions and limited access to socioeconomic resources are important leverage points. This “larger system” might also include temporal scales other than those included in the causal loop diagram. For example, changes in psychological processes at the individual level might, if accompanied by improved access to socioeconomic resources, eventually affect the initial conditions for the next generation.

By describing variables as part of feedback loops, we also take a step towards identifying whether intervening on a particular variable can have a balancing or reinforcing effect within the entire system, driving it towards a different trajectory. For example, if the necessary precondition that access to socioeconomic resources is improved is met, the amplifying feedback loop that underlies either the deterioration or the development of coping resources can serve as a leverage point within the system as it can act either as a vicious or a virtuous cycle. While we should not expect that access to coping resources is enough to break conditions of chronic stress, an intervention that supports problem approaching coping strategies to meet their objective may contribute to the restoration of access to coping resources. When coping resources such as mastery and self-esteem are built up in a specific state, this may translate to a global trait belief, which can then enable problem approaching coping strategies. Importantly, two of the five feedback loops that were distinguished are related to access to coping resources. If a particular variable plays a key role in several feedback loops, unveiling overlapping burdens, this may suggest that this variable is a potential leverage point within the system. Psychological interventions that aim to enhance resilience, which has shown to be affected by factors such as self-esteem, active coping, self-efficacy and optimism [74], may act upon this leverage point. In combination with improving access to socioeconomic resources, such an individual-level approach might then contribute to diminishing the dominance of certain mechanisms within the system.

In this paper, we described the mechanisms underlying the relationship between exposure to adverse socioeconomic conditions and chronic stress from a complexity science perspective, focusing on amplifying feedback loops across different scales. By taking this perspective, we aimed to contextualise the existing portrayal of these mechanisms in the literature by showing that they are interlinked and embedded in social structures. For example, coping resources are defined in the literature as “relatively stable individual differences” [34], where especially neuroticism is often referred to as a character trait resulting from inter-individual variation in “negative emotional response to threat, frustration, or loss” and typified by “irritability, anger, sadness, anxiety, worry, hostility, self-consciousness, and vulnerability” [47]. By demonstrating that those factors that we commonly refer to as “coping resources” are affected via mechanisms such as the repeated insolvability of stressors, we intended to contribute to seeing these differences in a new light. Similarly, as previously addressed by others [69], the negative connotations that accompany problem avoidance coping strategies in the literature, suggesting that they are maladaptive in all circumstances, should be reassessed. By describing the mechanisms that could evoke problem avoidance in a context of adverse socioeconomic conditions, we aimed to shift the focus away from the individual to the context. This is why, despite the connotations of victim blaming that the terms “coping resources” and “problem avoidance” may convey due to how they are currently used in the literature, we have used this terminology in the CLD.

Recognising that chronic stress is one of the interlocking pieces that can explain the effect of exposure to adverse socioeconomic conditions on mental health may be important for clinical practice, for example when treating patients with depressive symptoms in relation to chronic stress. The value of the CLD in this setting seems to lie in particular in that it contextualises individual-level psychological processes. It shows that these psychological processes are part of several interacting mechanisms that, in combination, result in chronic stress and are driven by a context of adverse socioeconomic conditions.

The CLD can also be expanded to include countervailing mechanisms, i.e. mechanisms that prevent people from developing chronic stress despite being exposed to adverse socioeconomic conditions. These countervailing mechanisms may in some cases serve to compensate for the feedback loop mechanisms leading to chronic stress, where through the principles of non-linearity a certain feedback loop may become dominant or remain inactive through small changes in the system. For example, if social support would be added to a person’s constellation of factors, this may result in the effect of the other causal mechanisms being counterbalanced for that specific person [75]. Extending the CLD with such mechanisms, as well as the interactions between socioeconomic resources (such as between money and knowledge [15]), would be a next step in obtaining a full understanding of why people exposed to adverse socioeconomic conditions more often suffer from chronic stress.

The CLD is subject to some methodological considerations. A CLD that was established on the basis of GMB should always be considered as a reflection of the collective understanding of a specific group of domain experts, in relation to a specific research question. In other words, a single, “true” CLD modelling “the dynamics driving chronic stress in a context of adverse socioeconomic conditions” cannot exist. More generally, it cannot be reiterated often enough that “all models are wrong” [76], as they represent an abstraction of reality meant as a useful medium that aids in understanding that reality. In line with this, the CLD should be seen as a summary of the current evidence base that is limited to the specific research question addressed in this paper. Nevertheless, to increase the structural validity, we substantiated the CLD by literature references and evaluated its structure by presenting it to eight independent domain experts who were not involved in the GMB process. Additional variables and causal links could be integrated into the CLD to further explicate the relationship between exposure to adverse socioeconomic conditions and chronic stress in more detail, using updated knowledge, under different contexts, for different research questions or reflecting the viewpoint of a different set of researchers. This highlights that a developed CLD may well be in continuous flux. Notwithstanding, we argue that at a qualitative level the present CLD contains the most prominent mechanisms that drive chronic stress in a context of adverse socioeconomic conditions which can at least serve to anticipate important non-linear behaviours.

We defined the boundaries and which variables were regarded as exogenous and as endogenous in relation to our research question, where different boundaries and a different role for particular variables may be called for when applying this CLD to another context. For example, we have taken access to socioeconomic resources as an initial condition that cannot be influenced from within the modelled system, whereas in fact this access is determined by social structures. For the purposes of the CLD, the distribution of socioeconomic resources is thus taken as a given, while this could actually be addressed in policy, as we have discussed above. On the other hand, while we recognise that the modelled system is evolving within a larger system, we do expect the mechanisms between the endogenous variables included in the CLD to be generalisable. For instance, even if a mechanism influencing socioeconomic resources would be included, we presume that the role of socioeconomic resources within the modelled causal links remains the same.

We have analysed and discussed the CLD’s feedback loops and hypothesised dynamics in a qualitative manner. In order get a more precise indication of the importance of the various mechanisms within the system ideally a computational model, such as a quantitative system dynamics model, would have to be developed based on the conceptual model (as we have previously realised in [77]). Related to this, even though we have made a first step by identifying the temporal scales that the variables and causal links act upon, the exact implications that the different durations of the modelled mechanisms eventually have on the system’s outcomes remain elusive until quantification is achievable. Nonetheless, we are convinced that a conceptual model does justice to the objectives of this paper.

## Conclusions

To conclude, we developed a CLD to apply a complexity science perspective to understanding the impact of exposure to adverse socioeconomic conditions on chronic stress, which might serve as a basis for developing novel hypotheses for future research on this topic. Taking a complexity science perspective clarifies that the social structures causing some people to be exposed to adverse socioeconomic conditions may sustain amplifying feedback loops with individual-level biological and psychological consequences. As such, we should take care not to conceptualise chronic stress as an individual-level issue but as the emergent outcome of a set of conditions. The CLD describes that exposure to adverse socioeconomic conditions implies recurrent stressor exposure which impacts chronic stress via amplifying feedback loops that together could be conceptualised as one vicious cycle. This means that in order for individual-level psychological interventions to be effective, the context of exposure to adverse socioeconomic conditions also needs to be addressed.

## Supplementary Information

**Additional file 1.** Causal links in table format. Overview of all causal links included in the causal loop diagram, each accompanied by the literature that forms the basis for a particular causal link.

## Data Availability

Not applicable.

## Abbreviations

CLD: Causal loop diagram
GMB: Group model building
SES: Socioeconomic status
