# Supplementary material for: Understanding the impact of exposure to adverse socioeconomic conditions on chronic stress from a complexity science perspective
Source: BMC Med. 2021 Oct 12;19:242. doi: 10.1186/s12916-021-02106-1 (PMC8507143; doi:10.1186/s12916-021-02106-1)
Supplement: Supplementary file 1 — Additional file 1. Causal links in table format. Overview of all causal links included in the causal loop diagram, each accompanied by the literature that forms the basis for a particular causal link. [file 12916_2021_2106_MOESM1_ESM.docx]

**Table S1 Causal links.**

| Causal link | | | Literature |
| --- | --- | --- | --- |
| *Section 1.1 Exogenous variables: initial conditions Uncontrollable childhood and life course stressors* | | | |
| CL1  CL2 | CL1: Stimulus 🡪 ~ Stressor  CL2: Primary appraisal of stimulus as stressor 🡪 ~ CL1 | Polarity link dependent on primary appraisal of stimulus:   - Stimulus & Primary appraisal of stimulus as a stressor (threat) 🡪 + Stressor - Stimulus & Primary appraisal of stimulus as not a stressor (challenge) 🡪 - Stressor | (Lazarus, 1991) |
| CL3 | Uncontrollable childhood and life course stressors 🡪 + Primary appraisal of stimulus as stressor | | (Epel et al., 2018) |
| CL4  CL5 | CL4: Uncontrollable childhood and life course stressors 🡪 - Coping resources  CL5: Socioeconomic resources 🡪 - CL4 | Strength link dependent on interaction between socioeconomic resources relative to stressor:   - If interaction socioeconomic resources sufficient to reappraise stressor, the effect of CL4 is less strong - If interaction socioeconomic resources insufficient to reappraise stressor, the effect of CL4 remains unchanged | CL4: (Bandura, 1994; Barlow, Ellard, et al., 2014; Galanakis et al., 2016; Gallo & Matthews, 2003; Juth et al., 2008; Luo et al., 2021; Riese et al., 2014; Robb et al., 2009; Taylor & Seeman, 1999; Wadsworth, 2015)  CL5: (Aldwin et al., 1996; Nurius et al., 2015) |
| *Section 1.2 Exogenous variables: initial conditions Socioeconomic resources* | | | |
| CL6 | Stressor 🡪 + Secondary appraisal of stressor as uncontrollable | | (American Psychological Organization, 2020; Lazarus, 1991; Smith & Kirby, 2009) |
| CL7 | Socioeconomic resources 🡪 - Secondary appraisal of stressor as uncontrollable | | (American Psychological Organization, 2020; Kivimäki et al., 2015; Lazarus, 1991; Smith & Kirby, 2009) |
| CL8 | Coping resources 🡪 - Secondary appraisal of stressor as uncontrollable | | (American Psychological Organization, 2020; Kivimäki et al., 2015; Lazarus, 1991; Smith & Kirby, 2009) |
| CL9 | Secondary appraisal of stressor as uncontrollable 🡪 + Stress response | | (Lazarus, 1991) |
| CL10 | Socioeconomic resources 🡪 + Solvability of stressors | | n/a |
| CL11 | Problem avoidance (relative to problem approaching) 🡪 - Solvability of stressors | | n/a |
| *Section 2.1 Endogenous variables: feedback loops Progressive deterioration of access to coping resources because of repeated insolvability of stressors* | | | |
| CL12 | Coping resources 🡪 - Problem avoidance (relative to problem approaching) | | (Bolger, 1990; Dumont & Provost, 1999; Lahey, 2009; Solberg Nes & Segerstrom, 2006; Stanisławski, 2019; Thoits, 1995) |
| CL13  CL14 | CL13: Problem avoidance (relative to problem approaching) 🡪 ~ Coping resources  CL14: Solvability of stressors 🡪 ~ CL13 | Polarity link dependent on solvability of stressors and problem avoidance:   - Solvable stressors & Problem avoidance 🡪 - Coping resources - Solvable stressors & Problem approaching 🡪 + Coping resources - Insolvable stressors & Problem avoidance 🡪 0 Coping resources - Insolvable stressors & Problem approaching 🡪 - Coping resources | CL13: (Aldwin et al., 1996; Barlow, Ellard, et al., 2014; Barlow, Sauer-Zavala, et al., 2014; Hecht, 2013)  CL14: (Belle & Doucet, 2003; Stanisławski, 2019; Wadsworth, 2015) |
| CL15  CL16 | CL15: Problem avoidance (relative to problem approaching) 🡪 ~ Stimulus  CL16: Solvability of stressors 🡪 ~ CL15 | Polarity link dependent on solvability of stressors and problem avoidance:   - Solvable stressors & Problem avoidance 🡪 0 Stimulus - Solvable stressors & Problem approaching 🡪 - Stimulus - Insolvable stressors & Problem avoidance 🡪 0 Stimulus - Insolvable stressors & Problem approaching 🡪 0 Stimulus | CL15: (Carver et al., 1989; Stanisławski, 2019)  CL16: (Carver et al., 1989; Stanisławski, 2019) |
| *Section 2.2 Endogenous variables: feedback loops Perception of stressors as uncontrollable due to learned helplessness* | | | |
| CL17 | Secondary appraisal of stressor as uncontrollable 🡪 + Problem avoidance (relative to problem approaching) | | (Contrada & Baum, 2011), (Seligman, 1972) |
| *Section 2.3 Endogenous variables: feedback loops Tax on cognitive bandwidth caused by the stress response and chronic stress* | | | |
| CL18 | Stress response 🡪 - Cognitive bandwidth under stress | | (Arnsten, 2009; Mani et al., 2013; Mullainathan & Shafir, 2013a, 2013b) |
| CL19 | Cognitive bandwidth under stress 🡪 - Problem avoidance (relative to problem approaching) | | (Arnsten, 2009; Mani et al., 2013; Mullainathan & Shafir, 2013a, 2013b) |
| *Section 2.4 Endogenous variables: feedback loops Stimulation of problem avoidance to provide relief from the stress response and free up cognitive bandwidth* | | | |
| CL20  CL21 | CL20: Problem avoidance (relative to problem approaching) 🡪 ~ Stress response  CL21: Solvability of stressors 🡪 ~ CL22 | Polarity link dependent on solvability of stressors and problem avoidance:   - Solvable stressors & Problem avoidance 🡪 - Stress response - Solvable stressors & Problem approaching 🡪 - Stress response - Insolvable stressors & Problem avoidance 🡪 - Stress response - Insolvable stressors & Problem approaching 🡪 + Stress response | CL20: (Stanisławski, 2019), (Taylor & Stanton, 2007)  CL21: (Stanisławski, 2019), (Taylor & Stanton, 2007) |
| *Section 2.5 Endogenous variables: feedback loops Susceptibility to appraising stimuli as stressors against a background of chronic stress* | | | |
| CL22 | Stress response 🡪 + Primary appraisal of stimulus as stressor | | (Epel et al., 2018) |

**References**

Aldwin, C. M., Sutton, K. J., & Lachman, M. (1996). The Development of Coping Resources in Adulthood. *Journal of Personality*, *64*(4), 837–871. https://doi.org/10.1111/j.1467-6494.1996.tb00946.x

American Psychological Organization. (2020). *Dictionary APA: secondary appraisal*. https://dictionary.apa.org/secondary-appraisal

Arnsten, A. F. T. (2009). Stress signalling pathways that impair prefrontal cortex structure and function. *Nature Reviews Neuroscience*, *10*(6), 410–422. https://doi.org/10.1038/nrn2648

Bandura, A. (1994). Self-Efficacy. In V. S. Ramachaudran (Ed.), *Encyclopedia of Human Behavior* (Vol. 4, pp. 71–81). Academic Press. (Reprinted in H. Friedman [Ed.], Encyclopedia of mental health. San Diego: Academic Press, 1998).

Barlow, D. H., Ellard, K. K., Sauer-Zavala, S., Bullis, J. R., & Carl, J. R. (2014). The Origins of Neuroticism. *Perspectives on Psychological Science*, *9*(5), 481–496. https://doi.org/10.1177/1745691614544528

Barlow, D. H., Sauer-Zavala, S., Carl, J. R., Bullis, J. R., & Ellard, K. K. (2014). The Nature, Diagnosis, and Treatment of Neuroticism. *Clinical Psychological Science*, *2*(3), 344–365. https://doi.org/10.1177/2167702613505532

Belle, D., & Doucet, J. (2003). Poverty, Inequality, and Discrimination as Sources of Depression Among U.S. Women. *Psychology of Women Quarterly*, *27*(2), 101–113. https://doi.org/10.1111/1471-6402.00090

Bolger, N. (1990). Coping as a personality process: A prospective study. *Journal of Personality and Social Psychology*, *59*(3), 525–537. https://doi.org/10.1037/0022-3514.59.3.525

Carver, C. S., Scheier, M. F., & Weintraub, J. K. (1989). Assessing coping strategies: A theoretically based approach. *Journal of Personality and Social Psychology*, *56*(2), 267–283. https://doi.org/10.1037/0022-3514.56.2.267

Contrada, R. J., & Baum, A. (2011). *The Handbook of Stress Science: Biology, Psychology, and Health*. Springer Publishing Company, LLC.

Dumont, M., & Provost, M. A. (1999). Resilience in Adolescents: Protective Role of Social Support, Coping Strategies, Self-Esteem, and Social Activities on Experience of Stress and Depression. *Journal of Youth and Adolescence*, *28*(3), 343–363. https://doi.org/10.1023/A:1021637011732

Epel, E. S., Crosswell, A. D., Mayer, S. E., Prather, A. A., Slavich, G. M., Puterman, E., & Mendes, W. B. (2018). More than a feeling: A unified view of stress measurement for population science. *Frontiers in Neuroendocrinology*, *49*(1), 146–169. https://doi.org/10.1016/j.yfrne.2018.03.001

Galanakis, M. J., Palaiologou, A., Patsi, G., Velegraki, I.-M., & Darviri, C. (2016). A Literature Review on the Connection between Stress and Self-Esteem. *Psychology*, *07*(05), 687–694. https://doi.org/10.4236/psych.2016.75071

Gallo, L. C., & Matthews, K. A. (2003). Understanding the association between socioeconomic status and physical health: Do negative emotions play a role? *Psychological Bulletin*, *129*(1), 10–51. https://doi.org/10.1037/0033-2909.129.1.10

Hecht, D. (2013). The Neural Basis of Optimism and Pessimism. *Experimental Neurobiology*, *22*(3), 173–199. https://doi.org/10.5607/en.2013.22.3.173

Juth, V., Smyth, J. M., & Santuzzi, A. M. (2008). How Do You Feel? *Journal of Health Psychology*, *13*(7), 884–894. https://doi.org/10.1177/1359105308095062

Kivimäki, M., Virtanen, M., Kawachi, I., Nyberg, S. T., Alfredsson, L., Batty, G. D., Bjorner, J. B., Borritz, M., Brunner, E. J., Burr, H., Dragano, N., Ferrie, J. E., Fransson, E. I., Hamer, M., Heikkilä, K., Knutsson, A., Koskenvuo, M., Madsen, I. E. H., Nielsen, M. L., … Jokela, M. (2015). Long working hours, socioeconomic status, and the risk of incident type 2 diabetes: a meta-analysis of published and unpublished data from 222 120 individuals. *The Lancet Diabetes & Endocrinology*, *3*(1), 27–34. https://doi.org/10.1016/S2213-8587(14)70178-0

Lahey, B. B. (2009). Public health significance of neuroticism. *American Psychologist*, *64*(4), 241–256. https://doi.org/10.1037/a0015309

Lazarus, R. S. (1991). Progress on a cognitive-motivational-relational theory of emotion. *American Psychologist*, *46*(8), 819–834. https://doi.org/10.1037/0003-066X.46.8.819

Luo, J., Zhang, B., & Roberts, B. W. (2021). Sensitization or inoculation: Investigating the effects of early adversity on personality traits and stress experiences in adulthood. *PLOS ONE*, *16*(4), e0248822. https://doi.org/10.1371/journal.pone.0248822

Mani, A., Mullainathan, S., Shafir, E., & Zhao, J. (2013). Poverty Impedes Cognitive Function. *Science*, *341*(6149), 976–980. https://doi.org/10.1126/science.1238041

Mullainathan, S., & Shafir, E. (2013a). *Scarcity: Why Having Too Little Means So Much*. Times Books, an imprint of Henry Holt and Company, LLC (North America), and Allen Lane (UK).

Mullainathan, S., & Shafir, E. (2013b). Freeing Up Intelligence. *Scientific American Mind*, *25*(1), 58–63. https://doi.org/10.1038/scientificamericanmind0114-58

Nurius, P. S., Green, S., Logan-Greene, P., & Borja, S. (2015). Life course pathways of adverse childhood experiences toward adult psychological well-being: A stress process analysis. *Child Abuse & Neglect*, *45*(3), 143–153. https://doi.org/10.1016/j.chiabu.2015.03.008

Riese, H., Snieder, H., Jeronimus, B. F., Korhonen, T., Rose, R. J., Kaprio, J., & Ormel, J. (2014). Timing of Stressful Life Events Affects Stability and Change of Neuroticism. *European Journal of Personality*, *28*(2), 193–200. https://doi.org/10.1002/per.1929

Robb, K. A., Simon, A. E., & Wardle, J. (2009). Socioeconomic Disparities in Optimism and Pessimism. *International Journal of Behavioral Medicine*, *16*(4), 331–338. https://doi.org/10.1007/s12529-008-9018-0

Seligman, M. E. P. (1972). Learned Helplessness. *Annual Review of Medicine*, *23*(1), 407–412. https://doi.org/10.1146/annurev.me.23.020172.002203

Smith, C. A., & Kirby, L. D. (2009). Secondary Appraisal. In D. Sander & K. R. Scherer (Eds.), *The Oxford Companion to Emotion and the Affective Sciences* (pp. 354–355). Oxford University Press. https://explorable.com/stress-and-cognitive-appraisal

Solberg Nes, L., & Segerstrom, S. C. (2006). Dispositional optimism and coping: A meta-analytic review. *Personality and Social Psychology Review*, *10*(3), 235–251. https://doi.org/10.1207/s15327957pspr1003_3

Stanisławski, K. (2019). The Coping Circumplex Model: An Integrative Model of the Structure of Coping With Stress. *Frontiers in Psychology*, *10*(MAR), 1–18. https://doi.org/10.3389/fpsyg.2019.00694

Taylor, S. E., & Seeman, T. E. (1999). Psychosocial Resources and the SES-Health Relationship. *Annals of the New York Academy of Sciences*, *896*(1), 210–225. https://doi.org/10.1111/j.1749-6632.1999.tb08117.x

Taylor, S. E., & Stanton, A. L. (2007). Coping Resources, Coping Processes, and Mental Health. *Annual Review of Clinical Psychology*, *3*(1), 377–401. https://doi.org/10.1146/annurev.clinpsy.3.022806.091520

Thoits, P. A. (1995). Stress, coping and social support processes: Where are we? Wat next? *Journal of Health and Social Behavior*, *Extra Issue*, 53–79.

Wadsworth, M. E. (2015). Development of Maladaptive Coping: A Functional Adaptation to Chronic, Uncontrollable Stress. *Child Development Perspectives*, *9*(2), 96–100. https://doi.org/10.1111/cdep.12112
